# Accidents by Lightning

**Published:** 1847-08

**Authors:** 


ARTICLE V.

ACCIDENTS BY LIGHTNING.

Injuries and deaths from lightning are of such frequent occurrence in this conntry as to render the subject worthy the attention of the profession. We hear of such cases occurring in different parts of the country every little while during the summer season, yet so few of them come under the observation of any one individual, that but little has been collated upon the subject. We have several interesting cases, but they are not sufficient of themselves to afford data for any general conclusions. To enable us to investigate the subject, we solicit, from our professional brethren, detailed histories of all such cases coming within their knowledge, of which they have reliable information; and if a sufficient collection of facts is made we will give the result of the inquiries we make, in the Journal.

The circumstances under which injuries have been received, the extent and symptoms, together with a detailed account of the treatment pursued, and its results, will be thankfully recceived. E.

